# Nurse managers’ leadership roles in mining primary healthcare settings in Gauteng

**DOI:** 10.4102/hsag.v27i0.1693

**Published:** 2022-02-08

**Authors:** Sanele E. Nene

**Affiliations:** 1 Department of Nursing, Faculty of Health Sciences, University of Johannesburg, Johannesburg, South Africa

**Keywords:** confusion, leadership roles, mining, nurse managers, primary healthcare

## Abstract

**Background:**

Nurse managers carry the light of leadership in mining primary healthcare settings (mPHCs). They must ensure that this light shines for their followers to improve the quality of service rendered in primary healthcare. However, the nurse managers in mPHCs are confusing their leadership roles with management roles. The existing policies such as operational management of mPHCs are also not clarifying the leadership roles of nurse managers.

**Aim:**

The purpose of this study was to understand the nurse managers’ leadership roles in mPHCs.

**Setting:**

This study was conducted in mPHCs, situated in Gauteng province, in the West Rand in 2017.

**Methods:**

A qualitative, exploratory, descriptive and contextual research design was employed in this study. The participants were selected using a non-probability purposive sampling method. Ten participants formed part of the study. To collect data, individual in-depth interviews were conducted. Giorgi’s descriptive thematic data analysis was used to analyse data. Measures to ensure trustworthiness and ethical principles were adhered to.

**Results:**

Three themes emanated: confusion of leadership roles with management roles, confusion of leadership roles with clinical roles and confusion of leadership roles with resources management roles.

**Conclusion:**

The importance of describing nurse managers’ leadership roles surfaced from this study to eradicate confusion.

**Contribution:**

The nurse managers in mPHCs are now understanding their leadership roles, and that leadership roles, management roles, clinical roles and resources management roles are distinguishable.

## Background

The ‘State of the World’s Nursing’ article published by the World Health Organization (WHO, 2020) confirms the importance of understanding leadership roles. The author, however, emphasises that nurse managers should more so understand the difference between leadership and management roles. It is a management facet of nurse managers to clearly demonstrate their leadership roles to use power and authority appropriately (Jooste 2017). The purpose of Alma Alta Primary Healthcare Declaration Theory of tackling health problems at the primary healthcare level to improve health for all people, calls for a clear description of leadership roles (Nene, Ally & Nkosi 2020; Rifkin 2018; WHO 1978).

Nurse managers as leaders in mining primary healthcare settings (mPHCs) are the role models to the unit managers and nurses (Ayers 2017; Williamson 2016). Lamb et al. (2018) elicited that nurse managers in mPHCs are expected to be both managers and leaders, but yet their leadership roles are not intertwined with their management roles.

In this context, nurse managers are employed by the mine to manage and lead the mPHCs, having unit managers and nurses reporting to them. In order to be a nurse manager in these settings, a registered nurse or general nurse must also hold an additional qualification in nursing management; therefore all the nurse managers have this additional qualification. Nurse managers report directly to the executive member of health and wellness, a senior vice president for the health and wellness portfolio. The mine built the PHCs on the site of each mine for easy access to employees and to create a heathy workforce; hence all health problems of mine employees that are at PHC level are managed in these settings. The services that are provided in these mPHCs are health promotion, medical, surgical, minor mine accidents, emergency services that are manageable at the primary healthcare level, tuberculosis (TB) and human immunodeficiency virus/acquired immunodeficiency syndrome (HIV/AIDS) management, maternal care and women health (Nene et al. 2020). Effective management of these services requires nurse managers to understand their leadership roles. All employees presenting with secondary and tertiary health problems are referred to designated nearest health establishments.

The management roles of nurse managers in mPHCs are to plan, organise, direct and control the activities to improve health for mine workers, and the leadership roles include inspiring, empowering, motivating and mentoring unit managers and nurses to achieve mPHCs objectives (Jooste 2017; Nene 2021). Nurse managers oversee the daily activities executed in a mPHC and influence how those activities are executed. Management and leadership roles have to be integrated in mPHCs to acquire operational efficiency and organisational effectiveness (Govender & Bussin 2020). However, this will be difficult to achieve when nurse managers’ leadership roles are confused with management roles (Breed, Downing & Ally 2020).

Nurses’ managers are regarding their leadership roles as their management roles, yet these are two different phenomena with unique attributes (Naidoo 2018; Udlis & Mancuso 2015). Clarification of leadership roles of nurse managers in mPHCs is an essential source of support, mentorship and role modelling because the success of any organisation depends on the quality of its leadership (Breed et al. 2020). In their study on mPHC leadership, Jooste and Hamani (2017) concluded that description and clarification of leadership roles should be a strategic exercise that enables nurse managers to become good and directional role models. Effective nurse managers are developed in a setting where their leadership roles are well understood (Jooste 2017).

### Problem statement

The author has observed that nurse managers are confusing their leadership roles with management roles; they lack clarity on what exactly are leadership roles. Nurse managers are not demonstrating attributes of leadership such as inspiration, motivation and mentorship; instead they focus mostly on their management roles: planning, organising, directing and controlling. For these nurse managers, it seems as if management roles and leadership roles are indistinguishable. However, confusion of leadership roles with management roles by the nurse managers in these mPHCs cannot be associated with incompetence because they all have an additional qualification in nursing management (South African Nursing Council, Regulation 635 of 2020). Existing policies in these mPHCs such as a policy on operational management for nurses and nurse managers are not clarifying the leadership roles of nurse managers, but it is only focusing on their management roles. The *Nursing Act* (33 of 2005) stipulates that a nurse manager with an additional qualification mentioned here should be competent to lead in any PHC. Confusion of leadership roles with management roles disconnects nurse managers from leading by example, empowering, mentoring, influencing others and transferring enthusiasm (Jooste 2017).

The background and problem statement discussed here triggered the following research question:

- What are nurse managers’ leadership roles in mPHCs in Gauteng?

### Purpose of the study

The purpose of the study was to understand the nurse managers’ leadership roles in mPHCs in Gauteng.

### Setting

This study was conducted in mPHCs, situated in Gauteng province in the West Rand. The mining company has built primary healthcare clinics in every site for easy access to its employees. These clinics are providing comprehensive primary healthcare services to more than 17 000 employees. Some of these clinics operate for 24 hours a day, and some only for 8 hours per day. There are 15 nurse managers employed as leaders in these clinics with more than 45 nursing personnel reporting to them.

## Research design and methods

A qualitative, exploratory, descriptive and contextual research design and a constructivistic paradigm were used in this study (Creswell & Creswell 2018). This design was deemed relevant for the study as it focuses on an understanding gained from naturalistic observations and exploration of reality, hence the design assisted to gain an understanding of the nurse managers’ leadership roles in mPHCs by following a systematic, interactive and holistic approach (Polit & Beck 2018).

### Population and sample

The population of this study consisted of all 15 nurse managers employed in mPHCs in the West Rand, in Gauteng. The sample of the study was 10 nurse managers who were purposively selected to assist to understand and describe the phenomenon (Creswell & Creswell 2018).

#### Inclusion and exclusion criteria

Inclusion criteria

- Nurse managers employed in mPHCs.
- With more than one years’ experience as a nurse manager in a mining sector as they should be familiar with the mPHC policies by then.

Exclusion criteria

- Nurse managers who were on leave during data collection were excluded from the study.

### Data collection

Individual in-depth semi-structured interviews were conducted to collect data in a systematic manner. Holloway and Galvin (2017) attested that individual interviews assist the participants to discuss their side of the story, as lived by them. The prospective participants were invited via email to an informative session, which was held in their workplace and those willing to participate in the study granted their permission in writing. The individual interviews were held in the offices of the participants and were audio-recorded to improve the quality of data collection, and permission for audio recording was also provided in writing by the participants. During the informative session, the research purpose, method and possible benefits of the study were made known to the prospective participants. The interviews were scheduled to suit the availability of each participant and were conducted at the workplace of the participants and lasted between 35 and 45 min. Data were collected from the 1st of September 2017 to the 18th of December 2017. Probing, reflecting and paraphrasing are the interactive communication strategies that were used during the interviews. The researcher is a qualified professional nurse and was a case manager in mPHC settings, reporting to the group case manager. The group case manager is reporting to the nurse managers. Hence, there was no direct relationship between the researcher and the participants. The following central question was asked to the participants of the study: What are your leadership roles in these mPHC settings? Data saturation was reached by the seventh participant, however, the researcher continued with the interviews until all 10 participants were interviewed.

### Data analysis

To analyse data, Giorgi’s four steps of descriptive data analysis method were used (Holloway & Gavin 2017). The author and an independent coder who is an experienced qualitative researcher individually perused and coded the transcripts and listened to the recordings, themes were recorded as they emanate using simple language descriptive words. A consensus meeting on findings was held with the independent coder.

### Measures to ensure trustworthiness

To ensure the trustworthiness of the study: credibility, transferability, dependability and confirmability were maintained according to Lincoln, Lynham and Guba (2011). Credibility was maintained using peer debriefing where the author had meaningful discussions on the study with two colleagues (Creswell & Creswell 2018). In-depth description of the research method and study context was carried out to ensure transferability. The dependability of the study was maintained by appointing a qualitative independent coder. Confirmability of the study was performed by reaching the consensus on the findings with the independent coder.

### Ethical considerations

The author followed four ethical considerations outlined by Dhai and McQuoid-Mason (2011): autonomy, justice, non-maleficence and beneficence. Ethical clearance was obtained from the Ethics Committee of the University of Johannesburg (reference number: REC-01-73-2017) and from the management of the organisation where the study was conducted. The informed consent to participate in the study and to conduct audio-recording was obtained from the nurse managers in mPHCs, after holding an information session on the study with them. The principle of autonomy and justice was upheld throughout the study by allowing the participants to participate voluntarily without risking prejudicial treatment and by treating the participants with respect and dignity (Polit & Beck 2018). Confidentiality and anonymity were maintained by using codes such as P2 during data collection and analysis process, transcripts and audio-recordings were locked away. Respect was demonstrated by calling the participants using their titles, and they were allowed to express their feelings in a way that is comfortable for them. No harm was imposed to the participants during the study and the findings of the study were communicated to the management of the organisation and to the participants.

## Findings

In this article, three themes are discussed, as they emerged from the study.

### Confusion of leadership roles with management roles

Nurse managers narrated their leadership roles as management roles. The participants are employed as nurse managers and are expected to manage the delivery of quality care in the mPHCs. This role also has elements of planning and monitoring. The participants appear to be oblivious to the fact that these are general management roles and not leadership roles. Affirming this are the following quotations from the participants:

‘My leadership role start[*s*] with planning … because if there is no plan that the team is following, everybody will [*go*] haywire.’ (P2, Female, 60 years)

‘My leadership role actually means from monitoring, even where I’m sitting I can actually see amongst the four provinces where our mining companies are.’ (P1, Male, 45 years)

Another participant mentioned that their leadership roles have more to do with management roles. This is confirmed by the following quotation:

‘Our leadership roles have more to do with management roles.’ (P2, Female, 50 years)

### Confusion of leadership roles with clinical roles

Nurse managers are overseeing the clinical roles and are also expected to assist with clinical roles such as treating the mine workers when the need arises, in the mPHCs. Nurse managers reported that their leadership roles are their clinical roles; this includes assessment, treatment and care and developing a programme on treatment of TB if it is identified as a problem. This is learnt from the participants’ statements indicated here:

‘One as the leader you even have to attend to clinical roles, which is assessment, treatment and care.’ (P7, Male, 59 years)

‘Your leadership role here you start by saying okay tuberculosis [*TB*] is my problem, if TB is my problem I am going to have a programme on treatment of TB.’ (P6, Female, 43 years)

Another participant alluded clinical auditing to their leadership role.

‘Well our leadership role is also clinical auditing, you don’t sleep at night when you have audits the following day.’ (P8, Female, 46 years)

### Confusion of leadership roles with resources management

Nurse managers in this context ensure that there is adequate availability of resources required for the delivery of quality care to the mine workers. When asked about their leadership roles, the participants mentioned resource management as their leadership role. The given quotations confirm this statement:

‘Obviously managing my staff is my leadership role, I need to check all of them.’ (P6, Female, 43 years)

‘As a leader, it is my leadership role to deal with limited stock and equipment issues.’ (P10, Female, 52 years)

Another participant put forth that financial management is their leadership role and that they have to account for all their financial activities directly or indirectly:

‘I will start with financial management it is my leadership role, and I need to account for all financial activities, directly or indirectly.’ (P4, Male, 38 years)

## Discussion of findings

Ten participants were interviewed during data collection, and their demographics are presented in Table 1.

**TABLE 1:** Demographics of the participants.

Variable	Value
**Gender**	
Men	4
Women	6
**Ethnicity**	
African	9
White	1
Age range	Between 38 and 60 years
**Total participants**	**10**

### Confusion of leadership roles with management roles

The participants mentioned their leadership roles as their management roles in these mPHCs, they allude planning and monitoring to leadership roles. Jooste (2017) attested that planning is the management role of a nurse manager and not a leadership role; however, these roles have to be merged to ensure that the vision of the organisation is reachable. Leadership roles should not be confused with management roles, as these two can be executed simultaneously, to ensure that the business of the day is performed accordingly (Liphadzi, Aigbavboa & Thwala 2016). Nurse managers have to demonstrate good leadership skills for them to be competent in their management roles, such as planning and monitoring (Naranjee, Ngxongo & Sibiya 2019). Gialamas et al. (2020) emphasised that leaders in mPHCs have to be able to distinguish between leadership roles and management roles for these two roles to rather be so integrated to improve client satisfaction.

Nurse managers have to lead the management role of monitoring to enhance accountability in mPHCs (Anderson et al. 2019). Lamb et al. (2018) and McFarlane (2020) affirmed that monitoring is a management role that assists to create accountable managers, and it is also developing these managers to become leaders who are reflective decision makers as it compels them to begin with an end in mind. Planning and monitoring form part of the management process (Jooste 2017); however, nurse managers are expected to demonstrate appropriate leadership attributes such as inspiration and motivation to implement a management process in mining settings.

Another participant stated that their leadership roles have more to do with management roles. Leadership roles are not management roles, but these two roles are distinct in their nature. Therefore, they have to complement each other for the achievement of organisational goals (Nene et al. 2020). Peiter, De Melo Lanzoni and De Oliveira (2016) affirmed that if nurse managers are not understanding their leadership roles, it is very simple for them to confuse such roles with their management roles.

### Confusion of leadership roles with clinical roles

The participants mentioned their leadership roles as clinical roles, however these roles are not identical. When asked about their leadership roles in mPHCs, they further stated that their leadership roles include assessment, treatment and care and developing a programme on treatment of TB. Clinical roles in PHC focus on enabling the opportunity to transform the care environment at the point of practice, it is based on clinical outcomes and nurse managers are leading the execution of clinical activities in mPHCs (Bender 2016). Gumbo (2017), and Jooste (2017) argued that leadership roles and clinical roles are two different facets and should not be reported as one key performance area. It is essential for nurse managers to have a clinical PHC experience for them to make relevant clinical decisions, such as the development of a programme on TB management. Tuberculosis is a leading fatal disease in the mines, and hence its management is a strategic priority but it is a clinical role, not a leadership role (World Bank 2019).

Govender, Proches and Kader (2018) posed a question that how can leadership roles on nurse managers be confused as clinical roles, whilst the quality of their leadership is being tested and strengthened in a clinical practice environment. Leaders of a clinical environment ensure that services are rendered enthusiastically and distinctively, leaving the client with no choice but to come back when the need arises. Nurse managers should not be deemed as generalists, clinicians and leaders, but their background of clinical practice should be abstracted and observable as they lead in such an environment (Heinrich 2017; Nelson 2017). It is therefore clinically unsound that the nurse manager leads a clinical environment such as PHC without an additional qualification in PHC relevant background (South African Nursing Council, Regulation 635 of 2020). This is postulated by the authors who stated that nursing is a practice-based discipline and ‘revolves around tenets of caring within an overarching altruistic framework within a specific setting’ (Jackson et al. 2009; Van Dam & Ford 2019).

One of the participants mentioned clinical auditing as one of the leadership roles. Efficient leadership improves the execution of clinical roles such as clinical auditing (Burns 2017). Lamb et al. (2018) stated that clinical auditing is a demanding clinical role that assists nurse managers to maintain quality service in mPHCs. According to Mullan (2016), clinical auditing is not a leadership role, but it is a tool used by nurse managers as leaders, to inspire their followers in order to identify poor performance or to celebrate excellence.

### Confusion of leadership roles with resources management roles

The participants reported that managing staff and dealing with limited stock and equipment are their leadership roles. Jooste (2017) posited that human resource management is a critical role for nurse managers, and it is executed and underpinned by the management process; planning, organising, directing and controlling. The leadership role of nurse managers is to encourage staff to take ownership towards the effective utilisation of limited stock and equipment in their respective settings (Stankiewicz, Seiler & Bortnowska 2017). Nene et al. (2020) attested that human resources management, stock and equipment management are all resources management roles, but the leadership role of nurse managers is to facilitate processes in place for the maximum use of available resources. Ayers (2017) affirmed that effective leadership is demonstrated when leaders are able to use the available resources efficiently to improve the performance of the organisation. Leadership roles should not be viewed as resources management roles, as this will enable nurse managers to apply their leadership roles such as effective communication when the health demands are increasing and when stretching the available resources is becoming too risky (Malelelo-Ndou, Ramathuba & Netshisaulu 2019; Nene 2021). Another resource management role of nurse managers is to ensure that the available resources are meeting the operational demands of mPHCs.

Confusion of leadership roles with resources management roles is a common mistake, it is envisaged that this is because nurse managers are mostly interested in resource management roles. It is, however, critical for nurse managers to master a resource management role of being effective using limited stock and equipment (Jooste 2017). Bai et al. (2017) added that nurse managers will not be available to demonstrate their leadership roles effectively, in an environment where stock and equipment are insufficient. Efficiency and the effectiveness of a leader are measured through the ability of coordinating the use of limited stock and equipment in the unit (Dania, Obro & Owhorhu 2016). Therefore, the leadership roles and resources management roles should be integrated to achieve the objectives of mPHCs.

Another participant mentioned that financial management is a leadership role that they have to account for. Udlis and Mancuso (2015) attested that financial management is a resource-related obligation for nurse managers who assist them to meet operational demands. Nurse managers have to account for all financial activities in their operations; however, this is not a leadership role (Govender et al. 2018).

### Recommendations

It is recommended from this study that understanding leadership roles is pivotal for nurse managers in mPHCs. This will enable nurse managers to distinguish between leadership roles and management, clinical and resources management roles. Available institutional policies and guidelines have to stipulate the leadership roles of nurse managers in mPHCs. The leadership roles of nurse managers have to be clearly outlined and discussed in all training programmes and in-service trainings for the nurse managers. A quantitative research measuring the understanding of nurse managers’ leadership roles in other PHC settings is encouraged.

### Limitations

The findings of this study could not be transferred, because it was qualitative and contextual in nature. Nurse managers are serving at the middle management level in mPHCs, and due to their hectic schedule they had limited time during the interviews. Consequently, they frequently lost concentration, but were however reconnected to the research question.

## Conclusion

The findings of the study revealed that nurse managers are confusing their leadership roles with management roles, clinical roles and resource management roles. The importance of describing the leadership roles of nurse managers surfaced to eradicate confusion. Policies and guidelines where leadership roles of nurse managers are stipulated should be developed.
